# In Situ Synthesis of a Bi_2_Te_3_-Nanosheet/Reduced-Graphene-Oxide Nanocomposite for Non-Enzymatic Electrochemical Dopamine Sensing

**DOI:** 10.3390/nano12122009

**Published:** 2022-06-10

**Authors:** Haishan Shen, Byungkwon Jang, Jiyoung Park, Hyung-jin Mun, Hong-Baek Cho, Yong-Ho Choa

**Affiliations:** Department of Materials Science and Chemical Engineering, Hanyang University, 55 Hanyangdaehak-ro, Sangnok-gu, Ansan 15588, Gyeonggi-do, Korea; seadheart@hanyang.ac.kr (H.S.); bkjang89@hanyang.ac.kr (B.J.); pjiyoung74@gmail.com (J.P.); brainbreak@hanyang.ac.kr (H.-j.M.); hongbaek@hanyang.ac.kr (H.-B.C.)

**Keywords:** non-enzymatic, electrochemical sensor, dopamine, Bi_2_Te_3_/rGO nanocomposite

## Abstract

Dopamine is a neurotransmitter that helps cells to transmit pulsed chemicals. Therefore, dopamine detection is crucial from the viewpoint of human health. Dopamine determination is typically achieved via chromatography, fluorescence, electrochemiluminescence, colorimetry, and enzyme-linked methods. However, most of these methods employ specific biological enzymes or involve complex detection processes. Therefore, non-enzymatic electrochemical sensors are attracting attention owing to their high sensitivity, speed, and simplicity. In this study, a simple one-step fabrication of a Bi_2_Te_3_-nanosheet/reduced-graphene-oxide (BT/rGO) nanocomposite was achieved using a hydrothermal method to modify electrodes for electrochemical dopamine detection. The combination of the BT nanosheets with the rGO surface was investigated by X-ray diffraction, X-ray photoelectron spectroscopy, field-emission scanning electron microscopy, transmission electron microscopy, Raman spectroscopy, and Fourier-transform infrared spectroscopy. Electrochemical impedance spectroscopy, cyclic voltammetry, and differential pulse voltammetry were performed to analyze the electrochemical-dopamine-detection characteristics of the BT/rGO nanocomposite. The BT/rGO-modified electrode exhibited higher catalytic activity for electrocatalytic oxidation of 100 µM dopamine (94.91 µA, 0.24 V) than that of the BT-modified (4.55 µA, 0.26 V), rGO-modified (13.24 µA, 0.23 V), and bare glassy carbon electrode (2.86 µA, 0.35 V); this was attributed to the synergistic effect of the electron transfer promoted by the highly conductive rGO and the large specific surface area/high charge-carrier mobility of the two-dimensional BT nanosheets. The BT/rGO-modified electrode showed a detection limit of 0.06 µM for dopamine in a linear range of 10–1000 µM. Additionally, it exhibited satisfactory reproducibility, stability, selectivity, and acceptable recovery in real samples.

## 1. Introduction

Dopamine is a neurotransmitter that plays a significant role in the central nervous, renal, cardiovascular, and hormonal systems [1]. High dopamine levels lead to cardiotoxicity, which includes symptoms, such as rapid heart rate, hypertension, heart failure, and drug addiction [2], whereas low dopamine levels may cause stress, Parkinson’s disease, Alzheimer’s disease, schizophrenia, and depression [2,3]. Thus, the rapid, simple, and efficient detection of dopamine is vital for understanding its biological functions and related biological processes and mechanisms [2].

Typical methods for the determination of dopamine include chromatography [4], fluorescence [5], electrochemiluminescence [6], colorimetry [7], and enzyme-linked techniques [8]. However, most of these methods are time-consuming, costly, and employ specific biological enzymes or complex detection processes. Therefore, non-enzymatic electrochemical methods are currently in the limelight because of their simplicity, rapidity, and high sensitivity [9,10].

In particular, a variety of nanomaterials, such as metals or metal oxides [11,12,13,14,15], polymers [16,17,18,19], and carbon materials [20,21], have been fabricated to modify bare glassy carbon electrodes (GCEs) and achieve excellent conductivity, sensitivity, and a large surface area.

Specifically, graphene-based materials, which are monolayer sp^2^-hybridized carbon atoms in a two-dimensional (2D) hexagonal framework, have a large theoretical surface area (2630 m^2^/g), excellent electrical conductivity (6.4 S/m), decent chemical and thermal stability, and a low production cost [22,23,24]. Additionally, they exhibit a wide potential window and excellent electrochemical characteristics, such as low charge-transfer resistance, excellent electrochemical activity, and rapid electron-transfer rate [23,24]. However, the water insolubility of graphene and its tendency to readily transform into graphite hinder its application as an electrochemical sensor [25,26].

Therefore, graphene oxide (GO) nanocomposites are employed in electrochemical biosensors to resolve these issues. In particular, the surface functional groups of GO, such as hydroxyl, carboxyl, and epoxy, can act as supporting substrates for metal/metal oxides or other nanomaterials to form nanocomposites and improve the electrochemical performance [27,28]. Moreover, reduced graphene oxide (rGO), which is derived from GO using chemical methods, is widely utilized for the synthesis of nanocomposites owing to its higher electrical conductivity compared with that of GO [29].

Bismuth telluride (Bi_2_Te_3_; BT) is extensively applied in photocatalysts [30], thermoelectric materials [31], photodetectors [32], gas sensors [33], capacitors [34], and electrochemical sensors [35,36,37] owing to its high ionic and electrical conductivities. The intrinsic layered crystal structure of BT can be readily synthesized using the solvothermal method to form 2D nanosheets. Additionally, the charge-carrier mobility on the thin 2D layers of metal chalcogenides with high crystallinity is significantly enhanced, and extremely thin nanosheets have been shown to act as topological insulators [38]. Thus, BT nanosheets drastically improve the direct electron transfer between the electrode surface and electrolyte in electrochemical sensors [35,36].

In this study, a non-enzymatic dopamine electrochemical sensor was constructed using high-conductivity rGO-supported BT nanosheets with a large surface area, which were synthesized using hydrothermal methods. The BT/rGO system exhibited advantages in terms of de-bundling rGO nanosheets and enabling their uniform growth into 2D nanosheets, thereby, effectively surpassing the electrochemical sensor. X-ray diffraction (XRD), X-ray photoelectron spectroscopy (XPS), field-emission scanning electron microscopy (FE-SEM), transmission electron microscopy (TEM), Raman spectroscopy, and Fourier-transform infrared (FTIR) spectroscopy results confirmed the successful fabrication of the BT/rGO nanocomposite.

Electrochemical impedance spectroscopy (EIS), cyclic voltammetry (CV), and differential pulse voltammetry (DPV) results showed that the BT/rGO-modified electrode exhibited enhanced electrochemical performance in terms of a low detection potential, high redox peak current, low limit of detection, wide linear range, and superior selectivity in the detection of dopamine. The unique structure of the BT/rGO nanocomposite can be exploited for use in other thermoelectric materials, photocatalysts, and different electrochemical sensors.

## 2. Experimental

### 2.1. Synthesis of BT/rGO Nanocomposite

**Figure 1:**
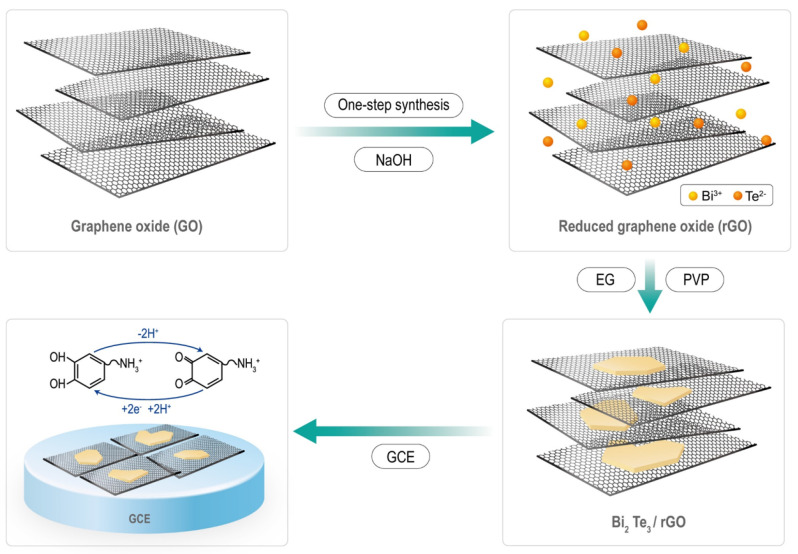
Synthesis of Bi_2_Te_3_ (BT)-nanosheet/rGO nanocomposite and the mechanism of electrochemical dopamine oxidation by the BT/rGO sensor.

A GO precursor was obtained from graphite powder using the improved Hummers’ method, as previously reported [39]. The BT/rGO nanocomposite was synthesized using a one-step in situ hydrothermal method (Figure 1). PVP (0.4 g; MW = 29,000, Sigma-Aldrich, Louis, US, Product Number (PN) #234257) and GO (40 mg) were completely dispersed in an ethylene glycol solution (18 mL) via ultrasonication for approximately 30–40 min. Bi_2_Oe_3_ (0.2330 g, Sigma-Aldrich, PN #223891), TeO_2_ (0.2394 g, Sigma-Aldrich, PN #243450), and NaOH (Sigma-Alrich, PN #S5881) solution (2 mL) was added to the preceding solution and vigorously stirred for 30 min. The resulting precursor was placed in a 30 mL Teflon-lined stainless-steel autoclave at 200 °C for 4 h. The final products were obtained by centrifugation and washed several times with ethanol and distilled water. The products were subsequently dried overnight under vacuum at 60 °C. BT and rGO were also similarly synthesized for comparison with the BT/rGO samples.

### 2.2. Preparation of Modified GCEs

A bare GCE (2 mm in diameter) was polished with alumina slurry (0.3 µm) and washed with ethanol and deionized (DI) water for 5 min under ultrasonication to remove pollutants. Subsequently, BT/rGO (5 mg) was dispersed in DI water (950 µL) via ultrasonication for 1 h to produce a homogenous dispersion, followed by the addition of Nafion (50 µL, Sigma Aldrich, PN #274704) to the suspension. A small amount of the solution (5 µL) from the suspension was added dropwise onto the GCE surface, and the resulting BT/rGO-modified working electrode was naturally dried at room temperature. The BT- and rGO-modified electrodes were similarly fabricated. The mechanism of dopamine oxidation by the designed sensor is illustrated in Figure 1.

### 2.3. Characterization

The crystal structure of the nanocomposite was analyzed by performing XRD (Cu Kα; Rigaku Co., Tokyo, Japan) at 40 kV and 100 mA. XPS (Al-Kα; Axis Nova, Shimadzu, Japan) was conducted to study the surface chemical composition and oxidation states, with the calibration performed using the C 1s binding energy at 284.6 eV. The surface morphology of the samples was observed by FE-SEM (Hitachi Ltd., Tokyo, Japan). High-resolution images and microstructure morphologies were obtained by TEM (JEM-2100F, JEOL, Tokyo, Japan) at 200 kV. Raman spectroscopy (UniRAM-3500, Micro Raman-PL sample chamber, Korea) was performed, particularly on the graphene-based compounds, to investigate the indirect structural characteristics of the carbon materials via their vibration dynamics. Moreover, FTIR spectroscopy (Nicolet iSTM 10, Thermo Fisher Scientific, Waltham, MA, USA) was conducted to analyze the functional groups.

### 2.4. Electrochemical Measurements

CV, DPV, and EIS analyses were performed using a standard three-electrode setup with the modified GCE, Ag/AgCl, and a Pt plate as the working, reference, and counter electrodes, respectively; a multichannel potentiostat/galvanostat/impedance analyzer (ZIVE MP 1, Wonatech, Seoul, Korea) was used for these experiments. EIS and CV measurements were conducted using K_3_Fe(CN)_6_ (5.0 mM, Sigma-Alrich, St. Louis, MI, USA, PN #702587) and K_4_Fe(CN)_6_ (5.0 mM, Sigma-Alrich, PN #P3289) dissolved in a KCl (0.1 M, Dae Jung, Siheung, Korea, PN #6566–4405) solution. Electrochemical measurements for the dopamine sensor were performed using a phosphate buffer solution (PBS; 0.1 M, Dae Jung, PN #6746-1100).

Glucose, ascorbic acid (Sigma-Alrich, PN #A92902), L-cysteine (Sigma-Alrich, PN #168149), calcium chloride (Sigma-Alrich, PN #C1016), sodium acetate (Sigma-Alrich, PN #236500), sodium citrate (Sigma-Alrich, PN #71498), urea (Sigma-Alrich, PN #U5128), and uric acid (Sigma-Alrich, PN #U2625) were used for interference analysis.

## 3. Results and Discussion

### 3.1. Characterization of BT/rGO

**Figure 2:**
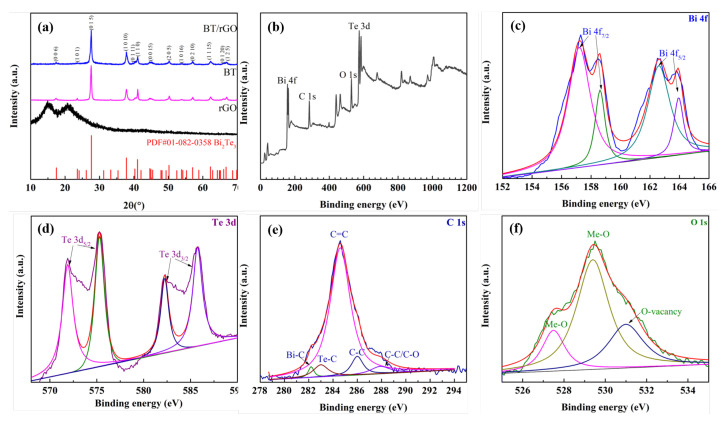
(**a**) XRD analysis of rGO, BT nanosheets, and BT/rGO. (**b**) XPS profile of BT/rGO. High-resolution (**c**) Bi 4f, (**d**) Te 3d, (**e**) C 1s, and (**f**) O 1s XPS profiles.

The XRD patterns of the bare BT and BT/rGO samples (Figure 2a) showed dominant diffraction peaks at 2θ values of 17.31°, 23.59°, 27.55°, 37.81°, 40.29°, 41.07°, 44.72°, 50.26°, 53.94°, 57.09°, 62.19°, 66.02°, and 66.86°, which approximately corresponded to the (0 0 6), (1 0 1), (0 1 5), (1 0 10), (0 1 11), (1 1 0), (0 0 15), (2 0 5), (1 0 16), (0 2 10), (1 1 15), (0 1 20), and (1 2 5) planes of the rhombohedral structure of BT (JCPDF, No. 01-082-0358) [40]. No peaks of rGO appeared owing to its low weight fraction, which is consistent with previously reported data [41,42].

XPS analysis, which was performed to study the oxidation states and surface chemical composition (Figure 2b), revealed the presence of four elements (Bi, Te, C, and O) in the BT/rGO composition. The Bi 4f XPS peaks at 157.2 and 162.6 eV (Figure 2c) approximately corresponded to the 4f_5/2_ and 4f_7/2_ binding energies, whereas the low-energy peaks at 158.6 and 164 eV were attributed to the oxidized bismuth-telluride layer (Bi–Te–O) [43,44]. The Te 3d_5/2_ and Te 3d_3/2_ XPS peaks appeared at 575.3 and 585.7 eV (Figure 2d), along with those of TeO_*x*_ at 572.0 and 582.2 eV [45].

The strong C 1s peak at 284.6 eV was attributed to the C=C bond of rGO, whereas the weak peaks at 286 and 287.9 eV corresponded to C–C and C–O/C=O (alkoxy/carbonyl) bonds, respectively, [46,47]; the peaks at 282.2 and 283 eV corresponded to Te–C [48,49] and Bi–C [50] bonds (Figure 2e).

The high-resolution O 1s peaks at 527.5 and 529.4 eV were attributed to metal–oxide bonds, indicating the presence of oxygen atoms [51,52,53], whereas the peak at 531 eV was ascribed to an oxygen vacancy present in the lattice (Figure 2f) [54,55]. Essentially, the XRD and XPS analyses showed that Bi and Te ions were easily absorbed and subsequently grew on the rGO surface to yield BT/rGO. The oxygen-containing functional groups of rGO (−OH and −COOH) are known to enable robust chemical bonding with transition metal ions [28,56].

**Figure 3:**
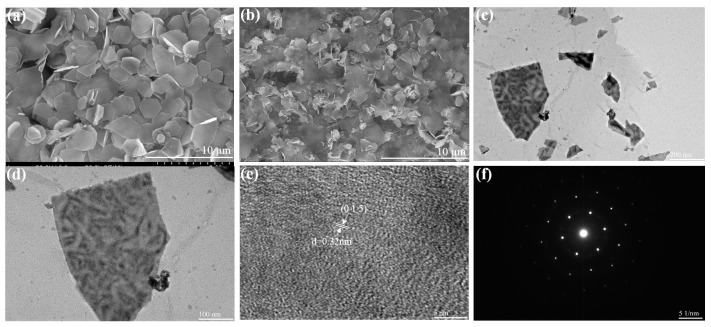
FE-SEM images of (**a**) BT and (**b**) BT/rGO. TEM images of (**c**) BT/rGO and (**d**) BT. (**e**) HRTEM image of BT/rGO. (**f**) SAED pattern of BT.

Furthermore, the surface morphology and structure of the BT and BT/rGO nanocomposites were analyzed by FE-SEM and TEM. The FE-SEM analysis (Figure 3a) revealed a two-dimensional (2D) morphology similar to that of bare BT nanosheets. The BT nanosheets with 2D morphology were uniformly embedded in the rGO network structure (Figure 3b), and displayed a sharp and flat surface.

Additionally, the TEM analysis (Figure 3c–f) corroborated the successful synthesis of the BT/rGO nanocomposite. The TEM image in Figure 3c indicates that certain randomly sized bare BT nanosheets grew on a thin layer of rGO sheets. The high-resolution image of the large nanosheets, which were approximately 500 nm in width (Figure 3d), revealed a highly crystallized lattice fringe with a spacing of 0.32 nm (Figure 3e), which was remarkably consistent with one of the main (0 1 5) planes of the rhombohedral structure of BT. The selected area electron diffraction (SAED) pattern (Figure 3f) showed a hexagonally symmetric diffraction spot pattern, indicating its single-crystalline characteristics.

**Figure 4:**
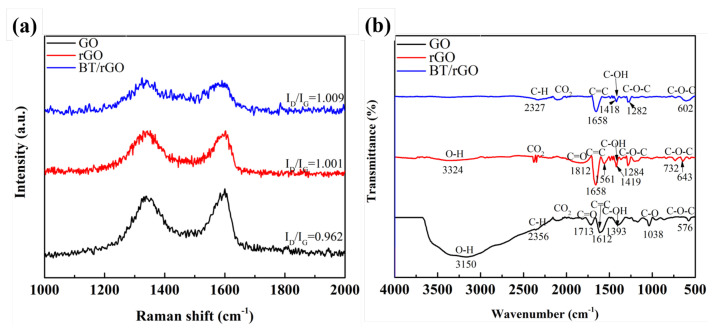
(**a**) Raman and (**b**) FTIR spectra of GO, rGO, and BT/rGO.

Raman spectroscopy is an important experimental technique for the characterization of carbon compounds, especially graphene-based materials; essentially, defects, phonons, and phonon–electron and electron–electron interactions can be determined and quantified, along with the number and orientation of graphene layers [57]. In the Raman spectra of the GO, rGO, and BT/rGO nanocomposites (Figure 4a), the D band at approximately 1350 cm^−1^ was assigned to the sp^3^-carbon-atom vibrations of disordered carbon and surface defects, and the G band at approximately 1580 cm^−1^ was associated with the in-plane vibration of the sp^2^ aromatic carbon structure, which indicates the symmetry and crystallization of carbon materials [57]. The relative intensity ratio between the D and G bands (I_*D*_/I_*G*_) indicates the degree of order or disorder of the carbon materials [57,58].

As of the reduction of GO, which involved the removal of oxygen-containing groups and preservation of its original layered structure, the intensity ratios of rGO and BT/rGO were higher than that of the GO sample. The intensity ratio corresponding to BT/rGO (1.009) was slightly higher than that of the rGO sample (1.001), which confirmed the slightly greater distortion of graphene by the embedding of BT nanosheets. In the FTIR spectra of GO, rGO, and BT/rGO, which were acquired in the 500–4000 cm^−1^ range to investigate the functional groups (Figure 4b), a broad band appeared at 3150 cm^−1^ in the GO spectrum, which was attributed to the O–H stretching vibration of the GO sheets [59].

Additionally, the peaks at 2356, 1713, 1612, 1393, 1038, and 576 cm^−1^ corresponded to the stretching vibrations of C–H, C=O, C=C, C–OH, C–O, and C–O–C, respectively [22,60]. The rGO peaks corresponding to the O–H and C=O bending at 3324 and 1812 cm^−1^, respectively, showed reduced intensities; moreover, the peak corresponding to C–H bonding disappeared, indicating the reduction of GO by NaOH. Moreover, the peaks corresponding to O–H disappeared, and certain peaks of the BT/rGO sample decreased in intensity compared to those of rGO, likely due to the BT nanosheets grown on the rGO surface.

### 3.2. EIS Analysis

**Figure 5:**
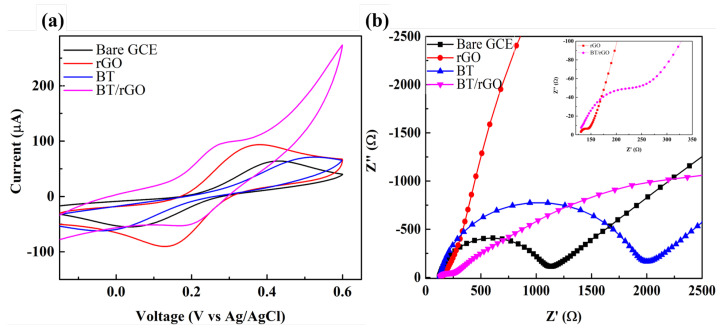
(**a**) CV and (**b**) Nyquist plots of bare GCE, GO, BT, and BT/rGO in KCl (0.1 M) with [Fe(CN)_6_]^3−/4−^ (5 mM). The inset in (**b**) shows an expanded view of EIS profiles of the rGO- and BT/rGO-modified electrodes.

The electrochemical properties of the modified GCE were determined using KCl (0.1 M) containing [Fe(CN)_6_]^3−/4−^ (5.0 mM). CV curves of [Fe(CN)_6_]^3−/4−^ were obtained using various modified GCEs at a scan rate of 100 mV/s (Figure 5a). BT/rGO clearly exhibited a higher redox peak current (98.58 µA) and lower redox potential (0.29 V) than those of the bare GCE (64.07 µA, 0.43 V), rGO-modified (93.72 µA, 0.38 V), and BT-modified (71.14 µA, 0.52 V) electrodes. For BT-modified electrodes, the redox current peak was higher than bare GCE; however, the redox potential was lower than bare GCE. This means the pristine BT only boosts the active surface area.

Nyquist plots of the bare GCE, rGO, BT, and BT/rGO systems were obtained from 10 Hz to 100 kHz (Figure 5b). The diameter of the semicircle, which represents the interfacial charge-transfer resistance (R_*ct*_), was considerably larger for the BT-modified electrode than that for the bare GCE owing to the poor conductivity of the randomly distributed BT. However, the rGO- and BT/rGO-modified electrodes had smaller semicircular diameters than that of the bare GCE.

In particular, the rGO-modified electrode displayed a significantly smaller semicircle (Figure 5b, inset) indicating the superior conductivity of rGO compared to that of the other electrodes. Owing to the high and low conductivities of rGO and BT, respectively, the R_*ct*_ values increased in the following order: rGO < BT/rGO < bare GCE < BT (150, 270, 1025, and 1870 Ω, respectively). These results highlight the potential of employing BT/rGO in electrochemical sensors based on its high electrical conductivity and sensitivity.

### 3.3. Electrochemical Behavior of Dopamine with Various Modified GCEs

**Figure 6:**
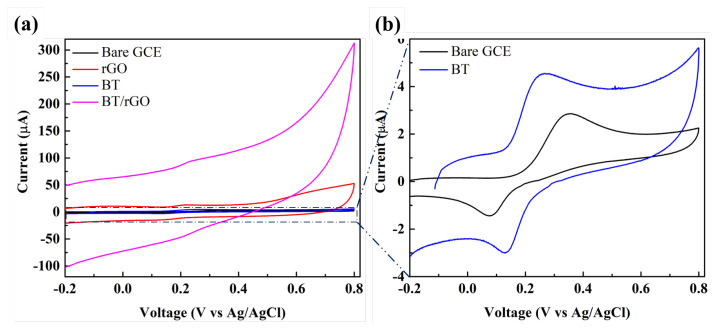
CV profiles of dopamine (100 µM) obtained using PBS (0.1 M) at a scan rate of 100 mV/s with (**a**) bare GCE, rGO, BT, and BT/rGO. (**b**) Magnified view of the bare GCE and BT electrode data.

The electrochemical behavior of dopamine on the bare GCE, rGO, BT, and BT/rGO electrodes was investigated by CV in PBS (0.1 M; pH = 7.03) containing dopamine (100 µM) at a scan rate of 100 mV/s. The bare GCE exhibited a CV response as an anodic peak current (I_*pa*_) at 2.86 µA corresponding to an anodic peak potential (E_*pa*_) of 0.35 V (Figure 6b; magnified version of Figure 6a). Moreover, BT exhibited I_*pa*_ and E_*pa*_ values of 4.55 µA and 0.26 V, respectively, whereas rGO showed the corresponding values at 13.24 µA and 0.23 V, respectively.

In particular, BT/rGO exhibited a pair of redox peaks at a higher I_*pa*_ (94.91 µA) and lower E_*pa*_ (0.24 V). The DPV results in Figure S1 for various modified electrodes also showed the same trends as in the CV results. The enhanced I_*pa*_ of BT/rGO was likely due to the synergistic effect of the decent electrical conductivity of rGO and the BT-nanosheet-enabled improvements in specific surface area and charge transfer. These results indicate the effectiveness of the BT/rGO nanocomposite as a non-enzymatic electrochemical sensor for dopamine.

### 3.4. Optimization of pH

**Figure 7:**
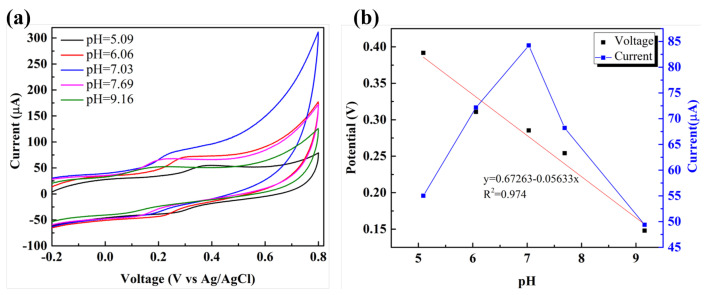
(**a**) CV profiles obtained at various pH values and (**b**) dependence of the oxidation peak potential and current on the various pH values for dopamine detection (100 µM) by BT/rGO at a scan rate of 100 mV/s.

The effects of pH on the electrochemical performance of BT/rGO were investigated by CV in PBS (0.1 M) containing dopamine (100 µM) at a scan rate of 100 mV/s (Figure 7). As the pH was varied from 5.09 to 9.16, Ipa increased to a maximum at a pH of 7.03, and subsequently decreased after a pH of 7.69. At low pH values, the oxidation of dopamine by releasing protons is difficult owing to the effect of H^+^ ions [61].

In contrast, at high pH value (>7.0), the absence of cation-*π* or electrostatic interaction between dopamine (NH_3_^+^) with graphene (COO^−^) induced lower current since dopamine solution changed to neutral form (pH 9.0, pKa1 = 8.87) [61,62]. Thus, PBS with a pH of 7.03 was employed as the optimal solution in the subsequent electrochemical experiments. However, the Epa values decreased with increasing pH in a linear manner, which could be expressed using the following linear regression equation:

(1) $$Epa\left(V\right)=0.672-0.0563\mathrm{pH}\left(R^{2}=0.974\right),$$

The slope corresponding to the aforementioned Equation (56.33 mV/pH) is close to the theoretical value provided by the Nernst Equation (59.2 mV/pH at 25 °C) [63], indicating that an equal number of electrons were transferred to protons in the electrochemical oxidation of dopamine.

### 3.5. Analytical Application of Sensor

**Figure 8:**
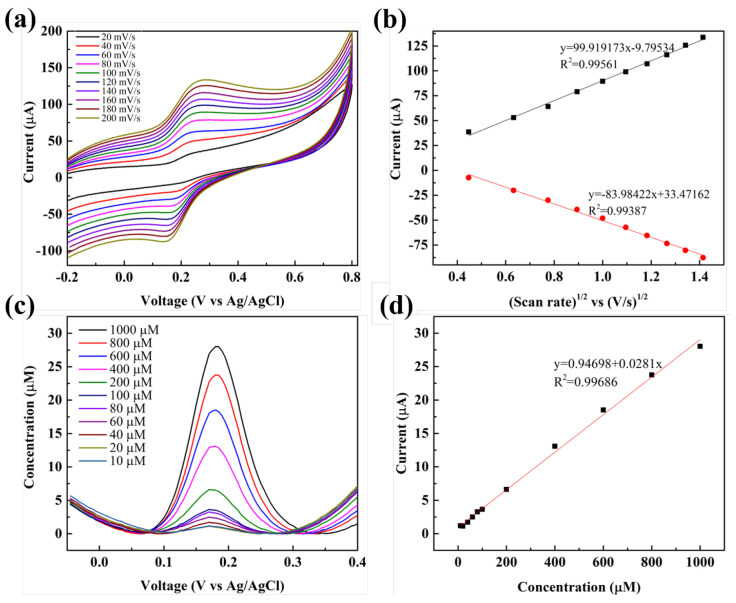
(**a**) CV curves obtained at different scan rates (20–200 mV/s) using BT/rGO for oxidation of dopamine (500 µM). (**b**) Calibration plots featuring the anodic (I_*pa*_) and cathodic peak currents (I_*pc*_) as a function of the square root of the scan rate. (**c**) DPV current responses for dopamine (10–1000 µM) in PBS (0.1 M, pH 7.03), and the (**d**) peak current–dopamine concentration plot.

Experiments were performed with PBS (0.1 M, pH 7.03) containing dopamine (500 µM) to determine the electrochemical characteristics of dopamine with respect to the BT/rGO electrode at different scan rates. Reversible CV curves of dopamine were acquired at scan rates of 20–200 mV/s (Figure 8a). The redox peak current corresponding to the reversible CV profiles progressively increased with increasing scan rate and slightly shifted to higher and lower potentials for the oxidation and reduction reactions, respectively.

Furthermore, a linear calibration plot between the redox peak current and scan rate was constructed based on the corresponding linear plot of I_*pa*_ vs. the square root of scan rate; the following linear regression equations were derived: I_*pa*_ (µA) = $99.919\sqrt{v}$ − 9.795 and I_*pc*_ (µA) = $-83.984\sqrt{v}$ + 33.471, where I_*pc*_ represents the cathodic peak (Figure 8b; corresponding correlation coefficients: 0.996 and 0.994, respectively). These results indicate that the electrochemical oxidation of dopamine at the BT/rGO-modified electrode occurred in a diffusion-controlled manner.

The DPV method offers a higher resolution and sensitivity than those of CV and is frequently used for quantitative analysis of dopamine at the BT/rGO-modified electrode; this method enables the determination of the electrode sensing range, sensitivity, and limit of detection (LOD) of electrochemical sensors [27].

The DPV sensitivity data for different concentrations of dopamine in PBS (0.1 M, pH 7.03) were obtained (Figure 8c). The oxidation peak current of dopamine markedly increased with an increase in its concentration from 10 to 1000 µM. Additionally, the corresponding calibration curve of the oxidation peak current as a function of dopamine concentration demonstrated excellent linearity, resulting in a linear equation of I_*pa*_ (µA) = 0.028C (µM) + 0.947 (R^2^ = 0.997).

Additionally, the LOD for dopamine was calculated to be 0.06 µM from the linear calibration plot based on the equation LOD = 3Sb/S, where Sb and S represent the standard deviation and slope, respectively, [35]. The sensitivity of this system was determined to be 222.93 µA·mM^−1^cm^−2^. Table 1 presents a comparison of the sensitivity, linear range, and LOD values of the BT/rGO-modified GCE with those of other graphene-based modified GCE electrodes used for dopamine sensing, such as bare GO and metal/rGO, metal-oxide/rGO, and polymer/rGO nanocomposites.

The BT/rGO-based dopamine sensor showed a wide range of linear detection (10–1000 µM) and the lowest LOD (0.06 µM) among the listed specimens (almost one-fifth of the next-lowest previously reported value). Furthermore, a comparison of rGO-based dopamine sensors that exhibit a wide linear range (20–1000 µM) reveals that the LOD reported herein was 1/40th that of previously reported data, confirming the superior performance of the BT/rGO-based composite.

**Figure 9:**
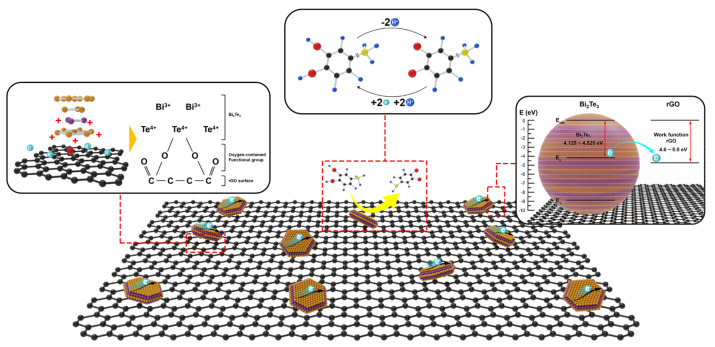
Schematic illustration of the charge transfer in the BT/rGO nanocomposite for the electrochemical sensing of dopamine.

Based on the dopamine-sensing performance of the BT/rGO nanocomposite, the related mechanism of electrochemical detection was elucidated (Figure 9). The oxygen-containing functional group of rGO enables strong ionic interactions with the Bi^3+^ or Te^4+^ of pristine 2D BT nanosheets to form a BT/rGO nanocomposite [66]. The 2D BT nanosheets implanted into an rGO matrix not only exhibit a large specific surface area but also act as a topological material with metallic electronic states at the boundary or surface [67].

These materials are highly mobile, and the delocalized surface states can promote interfacial charge transfer between the electrode surface and analyte, along with simultaneously enhancing the signal-to-noise ratio of the sensing current [37]. The electron affinity of BT and the work function of rGO have been estimated to be 4.125–4.525 eV [68] and 4.6–5.0 eV, respectively [69]. Owing to the band alignment between BT and rGO, a band-bending potential barrier is formed at their interface.

Electrons from the 2D nanosheets can be rapidly transferred to the rGO surface owing to the band barrier, thereby, boosting the electrical conductivity. These discussions indicate that the ultrahigh sensitivity of the BT/rGO nanocomposite in terms of its electrochemical behavior was primarily derived from the rapid charge-transfer interactions between the target dopamine molecules and BT/rGO-nanocomposite-modified electrode and the high conductivity of the BT/rGO nanocomposite.

### 3.6. Interference and Repeatability Analyses

**Figure 10:**
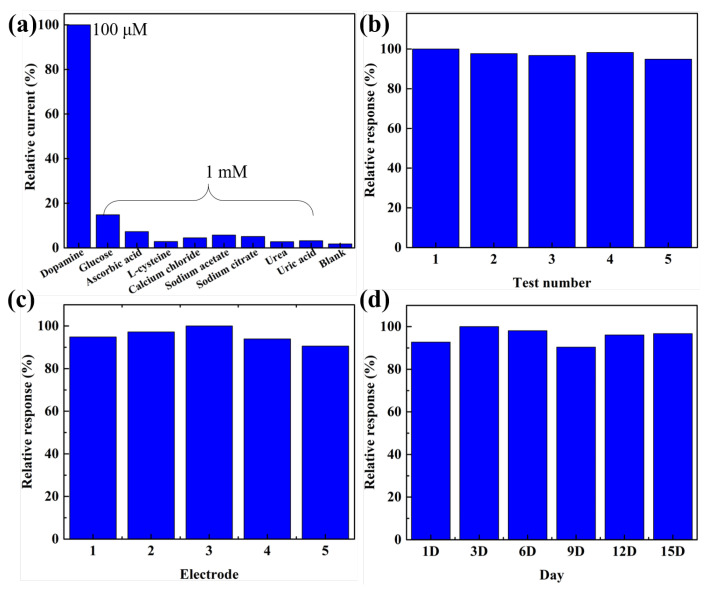
(**a**) Selectivity of the BT/rGO-modified electrode in the presence of dopamine (100 µM) and interfering materials (1 mM). (**b**) Repeatability, (**c**) reproducibility, and (**d**) storage stability analyses of the BT/rGO-modified GCE.

The selectivity of the BT/rGO-modified GCE was analyzed using different interferents, including glucose, ascorbic acid, L-cysteine, calcium chloride, sodium acetate, sodium citrate, urea, and uric acid, which are representative of common interfering compounds. No noticeable response was observed toward the other interferents even though the interfering materials (1 mM) were injected at a concentration 10× that of dopamine (100 µM; Figure 10a). The sensitivity toward dopamine, represented by the relative current (%), was almost six-times higher when reference molecules at identical concentrations were compared. This proved that the BT/rGO-modified electrode selectively responded to dopamine molecules.

Furthermore, the repeatability of the BT/rGO-modified electrode was investigated by performing three measurements using a single modified electrode in one day. The relative standard deviation (RSD) of the repeatability was calculated to be 1.89% (Figure 10b). The reproducibility of the BT/rGO-modified electrode was tested using five different modified electrodes (Figure 10c), which resulted in a decent reproducibility with 3.5% RSD. The storage stability of the BT/rGO-modified electrode was demonstrated using the long-term DPV method with dopamine (100 µM) in PBS (0.1 M, pH 7.03). After 15 d, the DPV signal of the dopamine biosensor was at 92% of its initial response signal, thereby, confirming its stability (Figure 10d).

### 3.7. Real Sample Analysis

Finally, the feasibility of the sensor was tested by injecting it with dopamine hydrochloride, which was diluted with PBS (0.1 M, pH 7.03) to provide a suitable concentration range for dopamine detection. After injecting dopamine hydrochloride at several concentrations, the current response was examined using the DPV method (Table 2), which yielded recoveries between 91.11% and 100.26%. Therefore, the fabricated BT/rGO-modified electrode showed acceptable practicability for real dopamine samples.

## 4. Conclusions

A rapid one-step synthesis of BT/rGO was demonstrated for a non-enzymatic electrochemical dopamine sensor. The analysis revealed that BT nanosheets and rGO were successfully combined to form nanocomposites. In terms of the surface morphologies of the composites, crystallized BT nanosheets grew uniformly on the surface of rGO nanosheets. The modification of glassy carbon electrodes (GCEs) with BT/rGO led to an outstanding electrocatalytic activity (94.91 µA, 0.24 V) for dopamine (100 µM), which was 7- to 33-times higher in terms of electrocatalytic oxidation than that of GCEs with BT (4.55 µA, 0.26 V), rGO (13.24 µA, 0.23 V), and bare GCE (2.86 µA, 0.35 V).

The EIS, CV, and DPV results revealed a low detection potential, high redox peak current, low limit of detection (0.06 µM), wide linear range (10–1000 µM), and superior selectivity (222.93 µA·mM^−1^cm^−2^) for dopamine owing to the synergic effects of the large specific surface area/high charge mobility of BT and the decent electrical conductivity of rGO. The obtained BT/rGO-modified electrode exhibited acceptable selectivity, stability, reproducibility, and repeatability. Additionally, the fabricated sensor was tested on real samples, and satisfactory results were obtained. The successfully combined BT/rGO nanocomposite is a promising candidate for thermoelectric materials, photocatalysts, and other electrochemical sensors.

## Figures and Tables

**Table 1:** Analytical parameters of dopamine oxidation at the BT/rGO electrode from this study compared with previously reported equivalents.

Modified Electrode	Technique	Sensitivity (µA·mM^−1^cm^−2^)	Linear Range (µM)	LOD (µM)	Ref.
3D porous GO	CV	-	0.1–30	1.28	[20]
rGO/CD ^1^	CV	-	0.5–20	1.41	[21]
Ag-rGO	DPV	110	10–70	1	[11]
Au-N-rGO ^2^	DPV	190	20–1000	2.4	[12]
CeO/rGO	DPV	-	10–150	2	[13]
MoS_2_-PANI/rGO	DPV	-	5–500	0.7	[64]
GQDs ^3^@MWCNTs ^4^	DPV	-	0.25–250	0.095	[65]
BT/rGO	DPV	222.93	10–1000	0.06	Present study

^1^ carbon dot; ^2^ n-doped rGO; ^3^ graphene quantum dots; and ^4^ multiwalled carbon nanotubes.

**Table 2:** Dopamine determination using the BT/rGO-modified GCE in injected dopamine hydrochloride.

Content (µM)	Detected (µM)	Recovered (%)	RSD (%)
10	9.11	91.11	7.8
240	237.62	99.01	2.85
480	481.25	100.26	3.91
560	556.99	99.46	4.6

## Data Availability

Not applicable.

## Supplementary Materials

The following supporting information can be downloaded at: https://www.mdpi.com/article/10.3390/nano12122009/s1, Figure S1: DPV of 100 µM dopamine in 0.1 M PBS at various modified electrode for bare GCE, rGO, Bi_2_Te_3_ (BT) and BT/rGO.
