# Supplementary material for: In Situ Synthesis of a Bi2Te3-Nanosheet/Reduced-Graphene-Oxide Nanocomposite for Non-Enzymatic Electrochemical Dopamine Sensing
Source: Nanomaterials (Basel). 2022 Jun 10;12(12):2009. doi: 10.3390/nano12122009 (PMC9228124; doi:10.3390/nano12122009)
Supplement: Supplementary file 1 [file nanomaterials-12-02009-s001.zip › nanomaterials-1758740-Supplementary Material.pdf]

Supplementary Material

In Situ Synthesis of a Bi_2Te_3-Nanosheet/Reduced-Graphene-Oxide Nanocomposite for Non-Enzymatic Electrochemical Dopamine Sensing

Haishan Shen, Byungkwon Jang, Jiyoung Park, Hyung-jin Mun, Hong-Baek Cho and Yong-Ho Choa*

Department of Materials Science and Chemical Engineering, Hanyang University, 55 Hanyangdaehak-ro, Sangnok-gu, Ansan, Gyeonggi-do 15588, Korea; seadheart@hanyang.ac.kr (H.S.); bkjang89@hanyang.ac.kr (B.J.); pjiyoung74@gmail.com (J.P.); brainbreak@hanyang.ac.kr (H.M.); hongbaek@hanyang.ac.kr (H.-B.C.)

* Correspondence: choa15@hanyang.ac.kr; Tel.:+82-31-400-5650

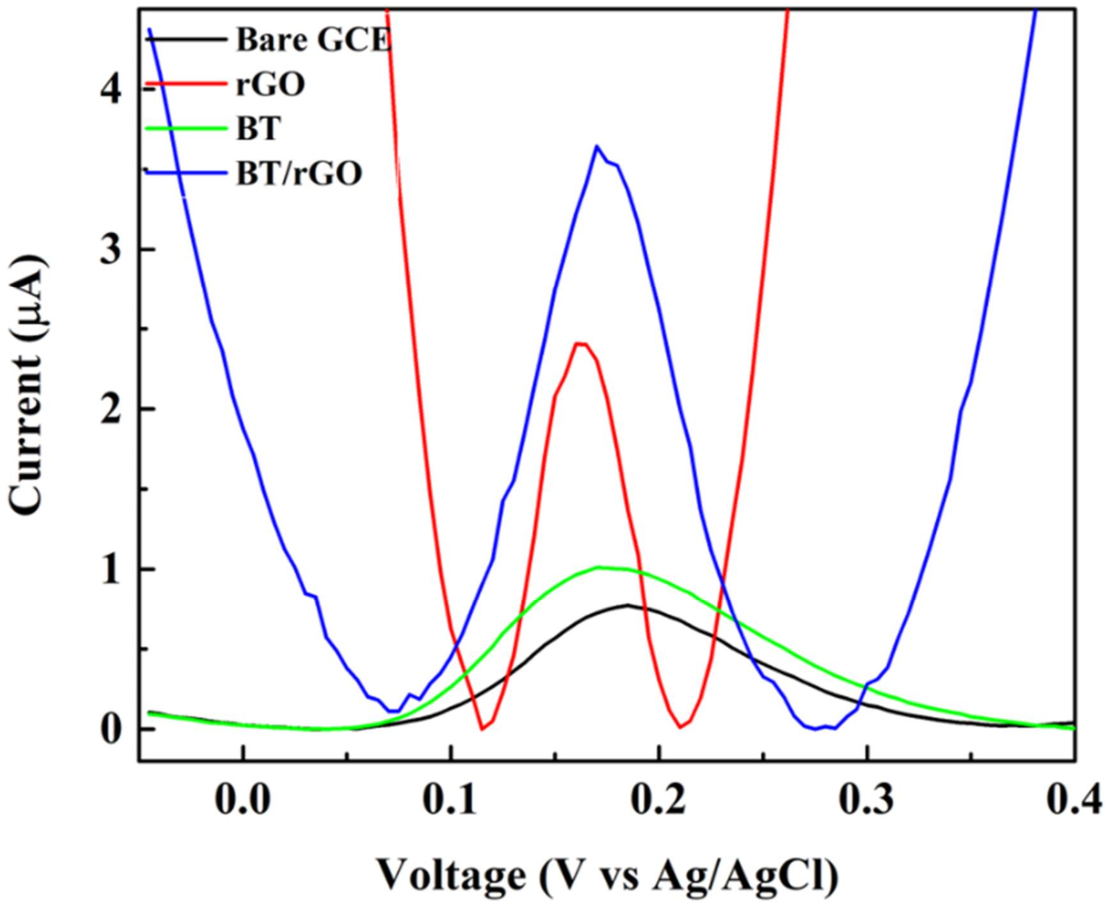

Figure S1. DPV of 100 μM dopamine in 0.1 M PBS at various modified electrode for bare GCE, rGO, Bi_2Te_3 (BT) and BT/rGO
